# *Pleurotus eryngii* Culture Filtrate and Aqueous Extracts Alleviate Aflatoxin B1 Synthesis

**DOI:** 10.1002/fsn3.70739

**Published:** 2025-10-19

**Authors:** Chahrazed Jaffali, Ayda Khadhri, Marzia Beccaccioli, Samira Aschi Smiti, Massimo Reverberi, Rosita Silvana Fratini, Slaven Zjalic

**Affiliations:** ^1^ University of El‐Manar, Faculty of Sciences, Plant, Soil and Environment Interactions Laboratory, Campus Academia Tunis Tunisia; ^2^ Department of Environmental Biology Sapienza University of Rome Roma Italy; ^3^ Department of Ecology, Agronomy and Aquaculture University of Zadar Zadar Croatia

**Keywords:** aflatoxin B1, aqueous extract, culture filtrate, *Pleurotus eryngii* var. *elaeoselini*, *Pleurotus eryngii* var. *ferulae*

## Abstract

Mycotoxins in food and feed are a significant health risk, even more so than pesticides and synthetic waste. These toxic secondary metabolites are produced by various fungal species, particularly after fungal colonization of crops. Aflatoxins produced mainly by *Aspergillus flavus* and *Aspergillus parasiticus* are among the most concerning mycotoxins. These fungi can colonize a range of crops, including maize and wheat, and produce aflatoxins both in the field and during post‐harvest. Aflatoxin B1 (AFB1) is the most toxic and carcinogenic, with demonstrated genotoxic, immunosuppressive, teratogenic, and hepatotoxic effects. Aflatoxins are stable in food and feed and can persist in the food chain, potentially appearing in milk as AFM1. Due to their toxicity, aflatoxins are strictly regulated globally, including in the European Union under Commission Regulation 2023/915. Climate change is increasing the frequency and concentration of mycotoxins in crops. The current control methods, including antifungals and synthetic chemicals, are ineffective and harmful, leading to the need for “greener” solutions. Recent research suggests that mushroom metabolites, particularly polysaccharides from species like *Pleurotus eryngii*, have potential in inhibiting aflatoxin synthesis. This study explores the effects of mycelial culture filtrates and aqueous extracts from two varieties of Tunisian *Pleurotus eryngii* on the growth and aflatoxin production of *Aspergillus flavus*.

## Graphical abstract

Pleurotus eryngii culture filtrate and aqueous extract could be a potent “greener” solution against Aflatoxin synthesis. The highest inhibition, 48.06%, is on the third day of incubation with the concentration of 0.5% of Pleurotus eryngii var. elaeoselini aqueous extract. Pleurotus eryngii var. ferulae culture filtrate at the concentrations of 0.5% and 1% exhibits the highest inhibition of aflatoxin synthesis, with 94%. Pleurotus eryngii var. elaeoselini culture filtrate and Pleurotus eryngii var. ferulae aqueous extract have the highest antioxidant activity.

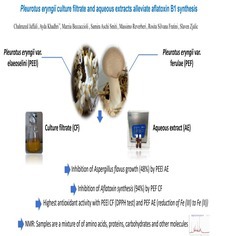

## 1 Introduction

The presence of mycotoxins in food and feed poses a major health risk to consumers, even higher than that of pesticides, food additives and plant toxins (Campos‐Avelar et al. 2021). Mycotoxins are secondary metabolites produced by a variety of fungal species that have toxic effects on vertebrates (Bennett and Klich 2003). They are synthesized after fungal colonization of the crop, for some genera such as *Fusarium* mainly in the field, or they can be produced both in the field and during the post‐harvest period, as is the case for some *Aspergillus* spp. (Bennett and Klich 2003). Owing to their toxicity, aflatoxins are among the mycotoxins of greatest concern to scientists and legislators alike. There are several *Aspergilus* species known as producers of aflatoxins, grouped in *Aspergillus* section Flavi, but *Aspergillus flavus* and *Aspergillus parasiticus* are considered the main culprits of aflatoxin occurrence in food and feed (Loncar et al. 2021, 2023). This mitosporic fungi are opportunistic plant pathogens that can colonize various vegetables, fruits and seeds, including cereals such as wheat and maize and synthetise aflatoxins both in pre and post‐harvest period (Agriopoulou et al. 2020). There are about 20 types of aflatoxins, of which only four, B1, B2, G1 and G2, are produced by fungi, while the others are the products of degradation and detoxification in the host organism. *Aspergillus flavus* is the main producer of AFB1 and aflatoxin B2 (AFB2) in maize. Aflatoxin B1 (AFB1) is the most common and most toxic (Corbu et al. 2023), it is classified by the IARC (1993) in Group 1A, carcinogenic to humans and animals, and its genotoxic, immunosuppressive, teratogenic and hepatotoxic effects have been demonstrated (Long 2020; Campos‐Avelar et al. 2021; Loncar et al. 2021). Aflatoxins are highly stable during feed and food processes and can also be transferred along the food chain unscathed or in (generally) less toxic metabolites such as AFM1 in milk. Therefore, the occurrence and concentration of aflatoxins in food, feed and food/feed stuff is limited by legislation worldwide (Loncar et al. 2021, 2023). In the European Union, it is regulated by Commission Regulation 2023/915 (EU, 2023). Climatic conditions drive the infestation of crops with mycotoxins and, therefore, the occurrence and concentration of mycotoxins in crops. Climate changes pave the way to increase both the frequency of occurrence of mycotoxins and their concentration in feed and food (Zjalic et al. 2024). The fight against aflatoxins in food and feed has long relied mainly on the use of antifungals and other synthetic chemicals, but from a food safety point of view, this approach is tantamount to “throwing oil on the fire” which can further exacerbate the problem rather than solve it. Hence the need to find “greener” solutions in this area is therefore worthwhile.

In the context of controlling aflatoxin synthesis, fungal metabolites, in particular fungal polysaccharides, have shown great potential (Reverberi et al. 2005; Zjalic et al. 2006; Scarpari et al. 2017; Loncar et al. 2023). In this paper, we test the effect of mycelial culture filtrates and aqueous extracts of carpophores of two varieties of Tunisian *Pleurotus eryngii* on the growth and biosynthesis of aflatoxin synthesized by *Aspergillus flavus*.

## 2 Material and Methods

### 2.1 *Pleurotus* Strains

**FIGURE 1:**
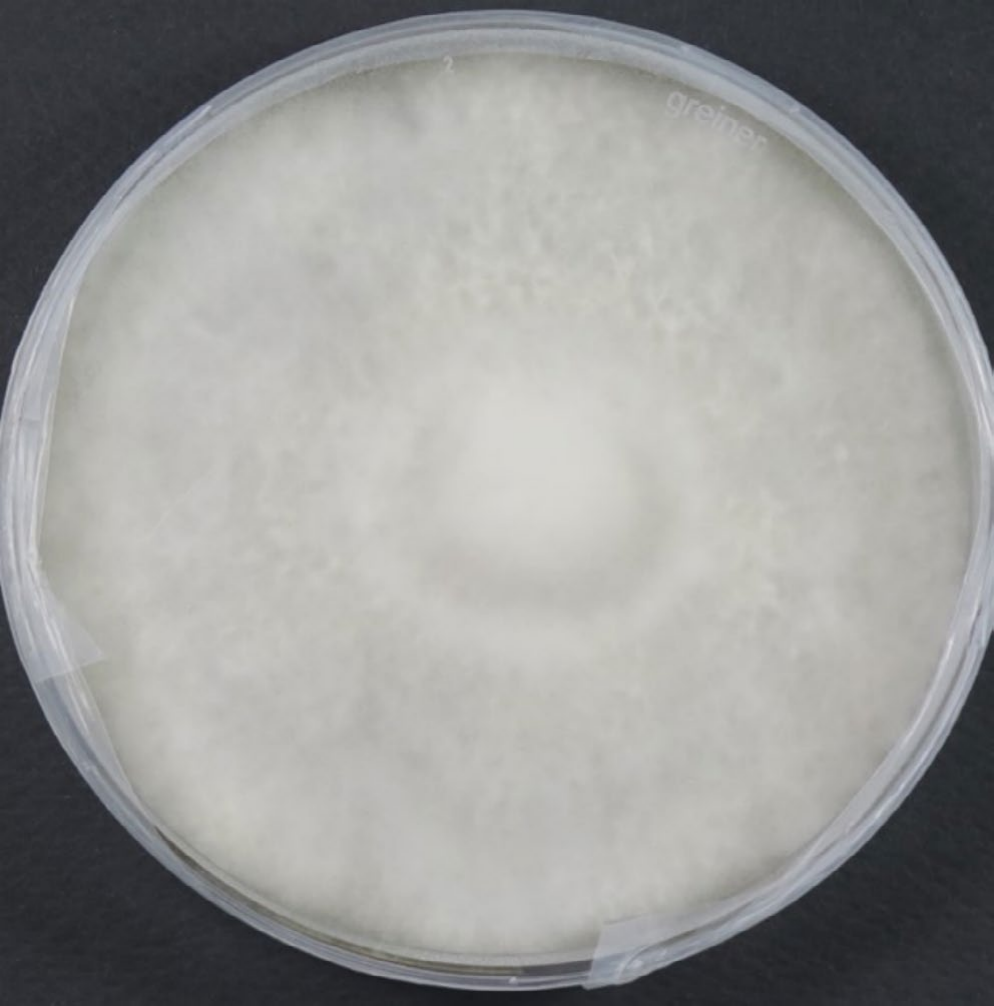
*Pleurotus eryngii* var. *ferulae* developed on potato dextrose agar (PDA).

Two varieties of *Pleurotus eryngii* were used in this study; they are collected from the Chela Morneg region in north Tunisia (36°41′40.4″N, 10°18′02.9″E). *Pleurotus eryngii* var. *elaeoselini* (PEEl) and *Pleurotus eryngii* var. *ferulae* (PEF) (DC.) Quél. A total of 1872 were cultivated on a mixed substrate of wheat straw (50%) and cardboard (50%) for PEEl, and wheat straw (75%), coffee grounds (25%), and wheat bran (5%) for PEF. Mycelium and naturally dried basidiocarps were conserved as dry fruiting bodies and as mycelia on Potato Dextrose Agar (PDA) medium at 4°C (Figure 1) at the Department of Biology, Faculty of Science, Tunis El Manar University, Tunis, Tunisia.

### 2.2 Pleurotus Aqueous Extracts

The aqueous extracts were prepared by mixing 10 g of homogenized powder of lyophilized fungi in 50 mL of boiling water (100°C). The mixture was then placed in an ultrasound bath at 25°C for 60 min (Sudha et al., 2016).

### 2.3 Preparation of Mycelial Culture Filtrate

Isolates are maintained on potato dextrose agar tubes at 4°C, and cultures are subcultured every 30 days. To obtain the inoculum for liquid cultures, mycelia from a tube were inoculated into 25 mL of sterilized potato dextrose liquid culture medium (PDB, Himedia) in 50 mL Erlenmeyer flasks (conical flasks) and incubated for 7 days at 25°C on a rotary shaker at 100 rpm. The liquid culture was homogenized under sterile conditions using a Waring 8012 blender (Waring, USA). Sterilized 1 L Erlenmeyer flasks containing 500 mL PDB were inoculated with 5% (v/v) of the homogenized mycelia and incubated for 14 days at 25°C under rotary shaking conditions (100 rpm). The mycelia were then separated from the culture filtrates by successive filtrations with rapid filters (Whatmann) to remove all mycelia. The resulting culture filtrates were freeze‐dried and used for further analysis (Scarpari et al. 2017; Loncar et al. 2023).

### 2.4 Collection of *Aspergillus flavus* Spores

*Aspergillus flavus* was cultured in potato broth medium (PDB) for 6 days at 28°C. The conidia were recovered by adding 10 mL of a sterile solution of water and Triton (0.01%) and the tube was vortexed for 2 min. Conidia suspension was filtered in sterile conditions, counted with a Thoma cell, and diluted to contain 1 × 10^5^ spores in 1 mL.

### 2.5 Samples Inoculation With Aspergillus Spores

**FIGURE 2:**
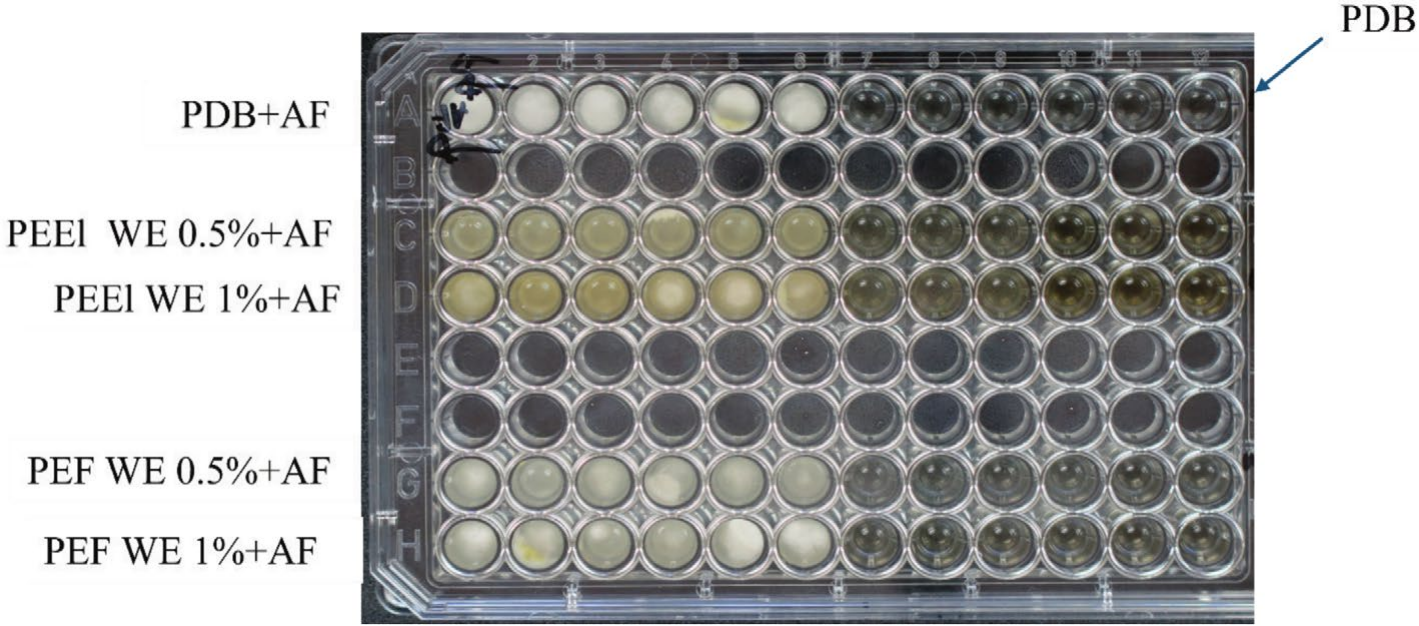
Inoculation of samples with *Aspergillus* spores using the multiwell plate.

Samples were prepared by dissolving freeze‐dried extracts in hot, sterile PDB at a rate that yielded 0.5% and 1% solutions (w/v) of each extract tested. Multiwell plates were used for the aflatoxin control test (Figure 2). A total of 200 μL of the sample served as a negative control, 190 μL of PDB + 10 μL of a solution containing 1 × 10^5^ conidia/mL as a positive control, and 190 μL + 10 μL of a solution containing 1 × 10^5^ conidia/mL as a test were added to the wells. The multiwell plate was incubated at 28°C for 7 days, and the absorbance was measured daily for the first 3 days. At the end, the plate was freeze‐dried to extract the mycotoxins. All tests were carried out in six replicates (6 wells).

### 2.6 Estimation of Fungal Growth of *A. flavus*

*Aspergillus flavus* growth is analyzed in the presence of a positive control (PDB + AF nutrient medium), culture filtrates, and aqueous extract of carpophores from two varieties of *Pleurotus eryngii*. The growth is monitored for 3 days by measuring absorbance at 620 nm of the plate (Torreggiani et al. 2023). The difference in absorbance is noted to assess the effect of aqueous extract and culture filtrate on *Aspergillus* growth, according to the following formula as follows:

ΔAbs=Absinoculated well−Abscontrol well

### 2.7 Extraction of Mycotoxins From Multi‐Well Plates

First, 1 mL of extraction solution was placed in 6 wells to resuspend the cultured fungus, then collected and placed in 2 mL Eppendorf. The extraction solution consisted of acetonitrile, water, and acetic acid, respectively in a 70:29:1 ratio, containing atrazine as a standard at a final concentration of 1 μM in 100 μL (final resuspension volume). The samples were vortexed for 20 min at 2500 rpm. They were then centrifuged for 10 min at room temperature at 10000 rpm. The supernatant was collected in a 2 mL Eppendorf tube, taking care not to collect the solid fraction at the bottom. The samples were dried with air at room temperature using a sample concentrator. After complete drying, the samples were resuspended in 100 μL of methanol and mixed for 5 min on a vortex mixer at minimum speed. They were then centrifuged at 13,000 rpm for 5 min to separate any solid impurities from the solution. Finally, 90 μL of solution was removed and placed in vials (Loncar et al. 2023). The concentration of aflatoxin B1 was determined by HPLC‐MS/MS (Agilent, Waldbronn, Germany) and recorded in ppb (Fanelli et al. 2000).

### 2.8 Mycelial Culture Filtrate and Aqueous Extracts Antioxidant Activity

The antioxidant activity was assessed by the 2,2‐diphenyl‐1‐picrylhydrazyl (DPPH) radical scavenging test and the reducing power of iron.

#### 2.8.1 DPPH Test

A 1 mL test sample of the extract at different concentrations was added in the presence of 250 μL of an ethanolic solution of DPPH. The mixture was left for 30 min at rest in the dark for incubation, and then the absorbance was measured at 517 nm using a spectrophotometer against a control (without extract) (Hatano et al. 1988). The results are expressed as a percentage of inhibition calculated following the decrease in color intensity of the mixture according to the following formula:

$$\mathrm{IP}\;\left(\%\right)=\left(A_{\text{control}}–A_{\text{extract}}/A_{\text{control}}\right)\times 100$$

By studying the variation in anti‐free radical activity as a function of extract concentration, the concentration corresponding to 50% inhibition (IC_50_) is determined; a low IC_50_ value corresponds to high efficacy of the extract.

#### 2.8.2 Iron's Reducing Power

The reducing activity of an extract is assessed by the redox reaction between the extract and transition metal ions, notably iron (Huang et al. 2005). Potassium ferricyanide K_3_ Fe (CN)_6_ supplies Fe^3+^ ions, which are reduced to Fe^2+^ by antioxidants present in the extract.

The reducing power was determined using the method described by Oyaizu (1986). This method involves mixing 1 mL of the extract at different concentrations with 2.5 mL of 0.2 M phosphate buffer at pH 6.6 and 2.5 mL of a 1% (w/v) K_3_Fe(CN)_6_ solution. The resulting mixture was incubated for 20 min at 50°C, then 2.5 mL of 10% trichloroacetic acid was added to stop the reaction. The mixture was centrifuged at 650 g for 10 min at room temperature, and 2.5 mL of the supernatant was added to 2.5 mL of distilled water and 0.5 mL of 0.1% (w/v) FeCl_3_. The absorbance is read at 700 nm against a blank in which the extract is replaced by the extraction buffer. The results are used to calculate effective concentration (EC_50_, mg/mL), the concentration of extract corresponding to an absorbance equal to 0.5. The EC_50_ value is obtained by interpolation of the linear regression curve (Mau et al. 2004).

### 2.9 NMR Analysis

NMR spectra were carried out on the Bruker AVANCE 300 MHz spectrometer at the Institut des sciences et technologies de l'environnement, Technoparc de Borj‐Cedria (ISSTE) in Tunisia. These spectra were recorded at room temperature (290 K), unless otherwise indicated. 1H NMR spectra are presented as follows: chemical shift (multiplicity, coupling constant, integration). The following abbreviations are used to indicate multiplicities: s, singlet; d, doublet; t, triplet; q, quartet; m, multiplet. Chemical shifts (δ) were given in parts per million (ppm) from tetramethylsilane with the solvent resonance as an internal standard in CDCl_3_ solution.

### 2.10 Statistical Analysis

The data were analyzed using XLSTAT 2021. Means were compared using the analysis of variance (ANOVA) test. Differences at the 5% threshold (*p* < 0.05) are considered statistically significant. Results are presented as mean ± standard deviation.

## 3 Results

### 3.1 Aqueous Extracts and Mycelial Culture Filtrates Yield

The yields of aqueous extracts (AE) from dry carpophores of PEF and PEEl are shown in Table 1, with yields of 34.29% and 31.2% for PEF and PEEl, respectively. Consequently, this difference is not significant (*p* > 0.05). For mycelial culture filtrates (CF), we note that the two varieties of oyster mushroom grown, PEF and PEEl, are twice as high as dried culture filtrate with yields of 62% and 67.2%, respectively, which were not significant.

**TABLE 1:** Yield of mycelial culture filtrate and aqueous extract.

		Mycelial culture filtrate (CF)	Aqueous extract (AE)
Yield	PEF	34.29^a^	62^a^
	PEEl	31.2^a^	67.2^a^

^a^ The values with the same superscript letters are not significantly different (*p* ≤ 0.05).

### 3.2 Analysis of *Aspergillus flavus* Growth Using a Multi‐Well Plate Assay

#### 3.2.1 Effect of Mycelium Culture Filtrates on the Growth of Aspergillus Flavus

**FIGURE 3:**
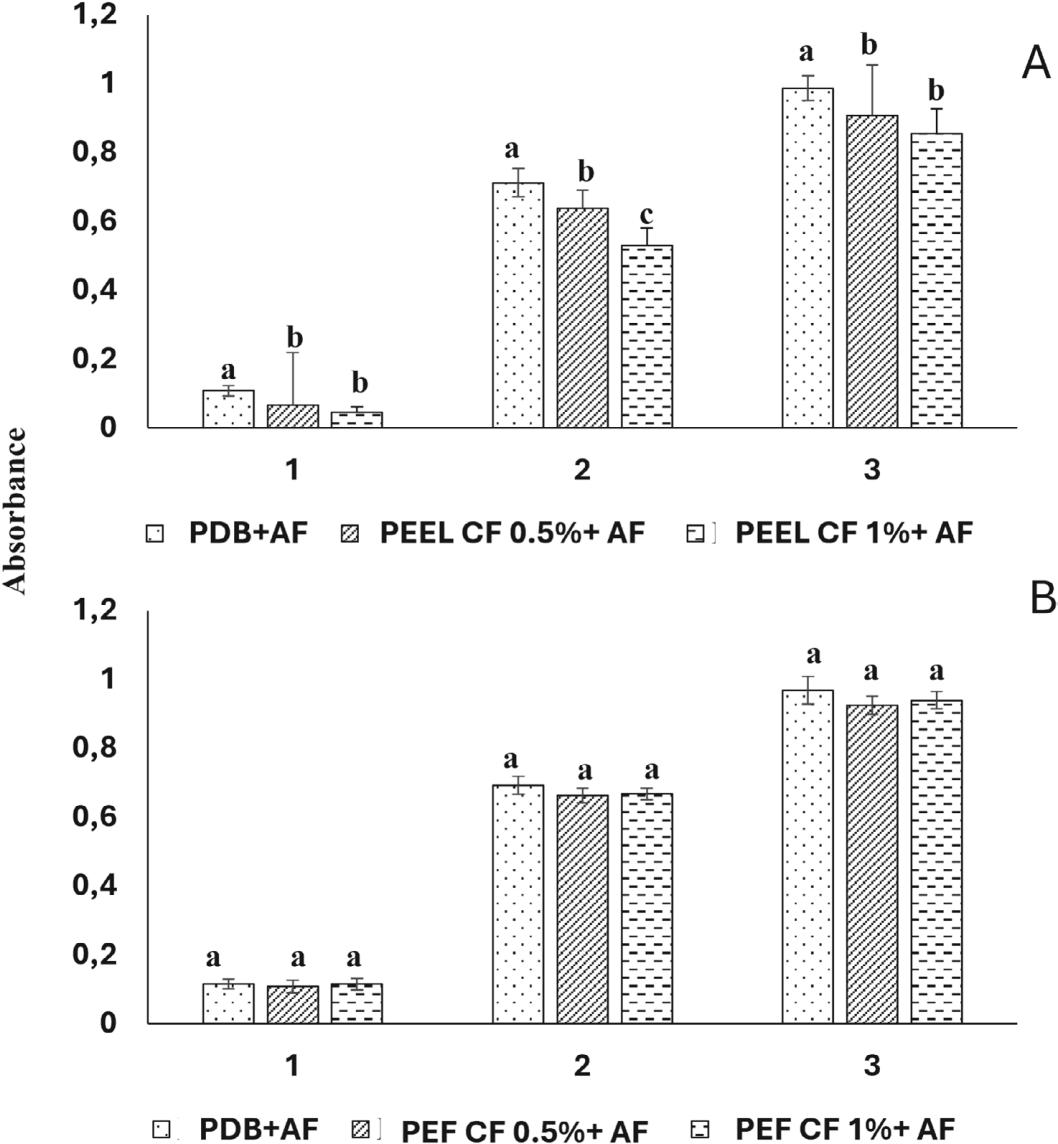
Variation of *Aspergillus flavus* growth with mycelial culture filtrate (CF) at 0.5% and 1% concentration of *Pleurotus eryngii* var. *elaeoselini* (A) and *Pleurotus eryngii* var. *ferulae* (B).

During the first 3 days of incubation, the growth of *A. flavus* was inhibited to some extent by both concentrations (0.5% and 1% w/v) of *P. eryngii* var. *elaeoselini* (PEEl) mycelial culture filtrate (CF). The highest inhibition rate, 25.35%, was observed on the second day of incubation (Figure 3A). The culture filtrate of *P. eryngii* var. *ferulae* (PEF) showed no significant effect on the growth of *A. flavus* (Figure 3B).

#### 3.2.2 Effect of Carpophores Aqueous Extract on the Growth of *Aspergillus flavus*

**FIGURE 4:**
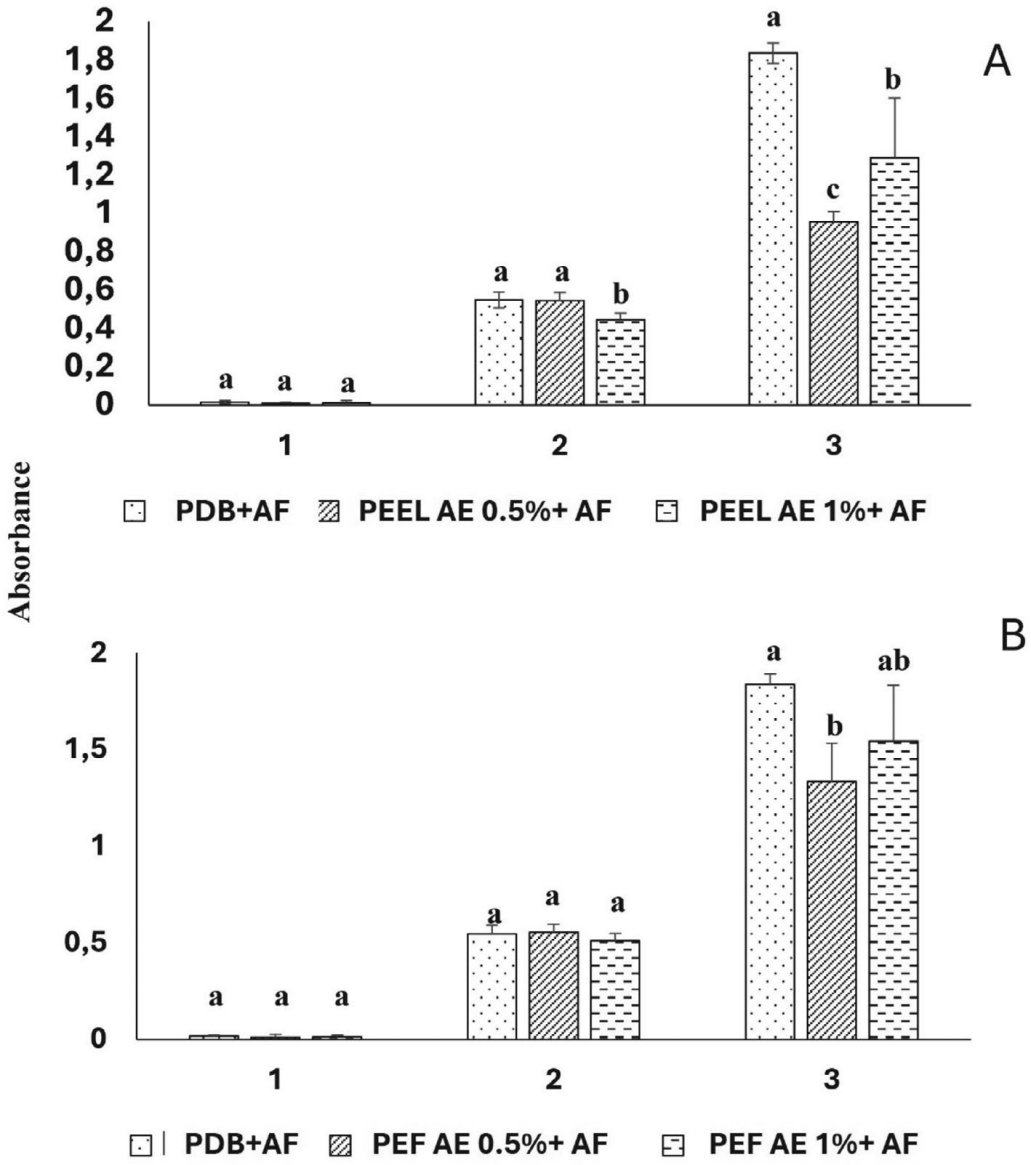
Effect of aqueous extract (AE) on the growth of *Aspergillus flavus* of *Pleurotus eryngii* var. *elaeoselini* (A) and *Pleurotus eryngii* var. *ferulae* (B).

The growth of *A. flavus* in the presence of two aqueous extracts (AE) of the two carpophores of PEEl and PEF is shown in Figure 4. The 1% w/v AE of PEEl significantly inhibited the growth of *A. flavus* by 18.42% (*p* < 0.05) on the second day of incubation compared to the control. This growth inhibition is even higher on the third day of incubation and is observed for both concentrations 0.5% w/v (48.06% inhibition) and 1% w/v (29.75% inhibition). In the case of PEF, it was found to have no effect on growth on the second day, while the AE at 0.5% w/v significantly reduced the growth of *A. flavus* by 27.35% on the third day (Figure 4B).

### 3.3 Culture Filtrates and Aqueous Extracts Effects on Aflatoxin Synthesis

The effects of aqueous extract (AE) and culture filtrate (CF) of PEEL and PEF are shown in Table 2. Both concentrations of CF (0.5% and 1% w/v) of both *P. eryngii* varieties significantly inhibited aflatoxin synthesis by *A. flavus* (*p* < 0.05). The PEF variety culture filtrates showed a higher inhibition; at both concentrations the inhibition was ~94%. The filtrate of the PEEL mycelial culture inhibited aflatoxin synthesis by ~87% (concentration 0.5% w/v) and ~67% (concentration 1% w/v).

**TABLE 2:** Variation of synthesized aflatoxin B1 quantity (%) in the presence of mycelial culture filtrate and aqueous extract.

	*Pleurotus eryngii*	Concentrations	Quantity of aflatoxin B1 (%)	Inhibition percentage compared with control (PDB + AF) (%)
Mycelial culture filtrate	PEEL	0.5%	13.04	86.95
		1%	32.69	67.3
	PEF	0.5%	5.43	94.58
		1%	5.44	94.56
Basidiocarps aqueuos extract	PEEL	0.5%	24	76
		1%	15	85
	PEF	0.5%	155	—
		1%	328	—

The aqueous extract from the fruiting bodies (AE) of PEEl significantly reduced aflatoxin synthesis by *A. flavus* at a concentration of 1% and 0.5% w/v by 85% and 76%, respectively. In contrast, the AE of the PEF variety led to a stimulation of aflatoxin synthesis compared to PDB at both concentrations.

### 3.4 Evaluation of Antioxidant Activity of Culture Filtrates and Aqueous Extracts

#### 3.4.1 DPPH Free Radicals' Activity

The results from the radical scavenging assays for both the mycelial culture filtrate and aqueous extract are summarized in Table 3, where inhibition concentration 50 (IC_50_) and efficiency concentration (EC_50_) values are presented. The IC_50_ represents the amount of extract required to scavenge 50% of the DPPH (2,2‐diphenyl‐1‐picrylhydrazyl) free radicals present in the solution. The most notable results were with *Pleurotus eryngii* var. *elaeoselini* culture filtrate with the lowest IC_50_ = 0.54 mg/mL followed by its aqueous extract with IC_50_ = 0.87 mg/mL. These results suggest that *Pleurotus eryngii* var. *elaeoselini* has a powerful ability to neutralize free radicals, with the culture filtrate showing the highest antioxidant efficiency.

**TABLE 3:** Mycelial culture filtrate and aqueous extract antioxidant activity determination by DPPH test and ferric reducing test.

		Antioxidant activity	Ferric reducing test EC (mg/mL)
		DPPH test IC_50_ (mg/mL)	
Mycelium Culture filtrate	PEF	1.71 ± 0.01	0.73 ± 0.05
	PEEl	0.54 ± 0.00	0.26 ± 0.08
Aqueous extract	PEF	1.05 ± 0.03	0.17 ± 0.04
	PEEl	0.87 ± 0.02	0.59 ± 0.07

#### 3.4.2 Antioxidant Activity Assessed Through the Reduction of Fe (III) to Fe (II)

The Fe (III) to Fe (II) reduction test evaluates the reducing power of a compound or extract, indicating its capacity to donate electrons. To assess the electron‐donating ability of culture filtrates and aqueous extracts from various *Pleurotus eryngii* varieties, we investigated their capacity to reduce Fe (III). The results of the Fe (III) reduction are summarized in Table 3. The results indicate that the aqueous extract of *P. eryngii* var. *ferulae* can donate electrons, suggesting its potential to scavenge free radicals. The highest activities were observed with the EC = 0.17 mg/mL, followed by the culture filtrate of *P. eryngii* var. *elaeoselini* (EC = 0.26 mg/mL) and the aqueous extract of *P. eryngii* var. *elaeoselini* (EC = 0.59 mg/mL).

### 3.5 NMR Analysis of PEEl and PEF Culture Filtrate and Water Extracts

**FIGURE 5:**
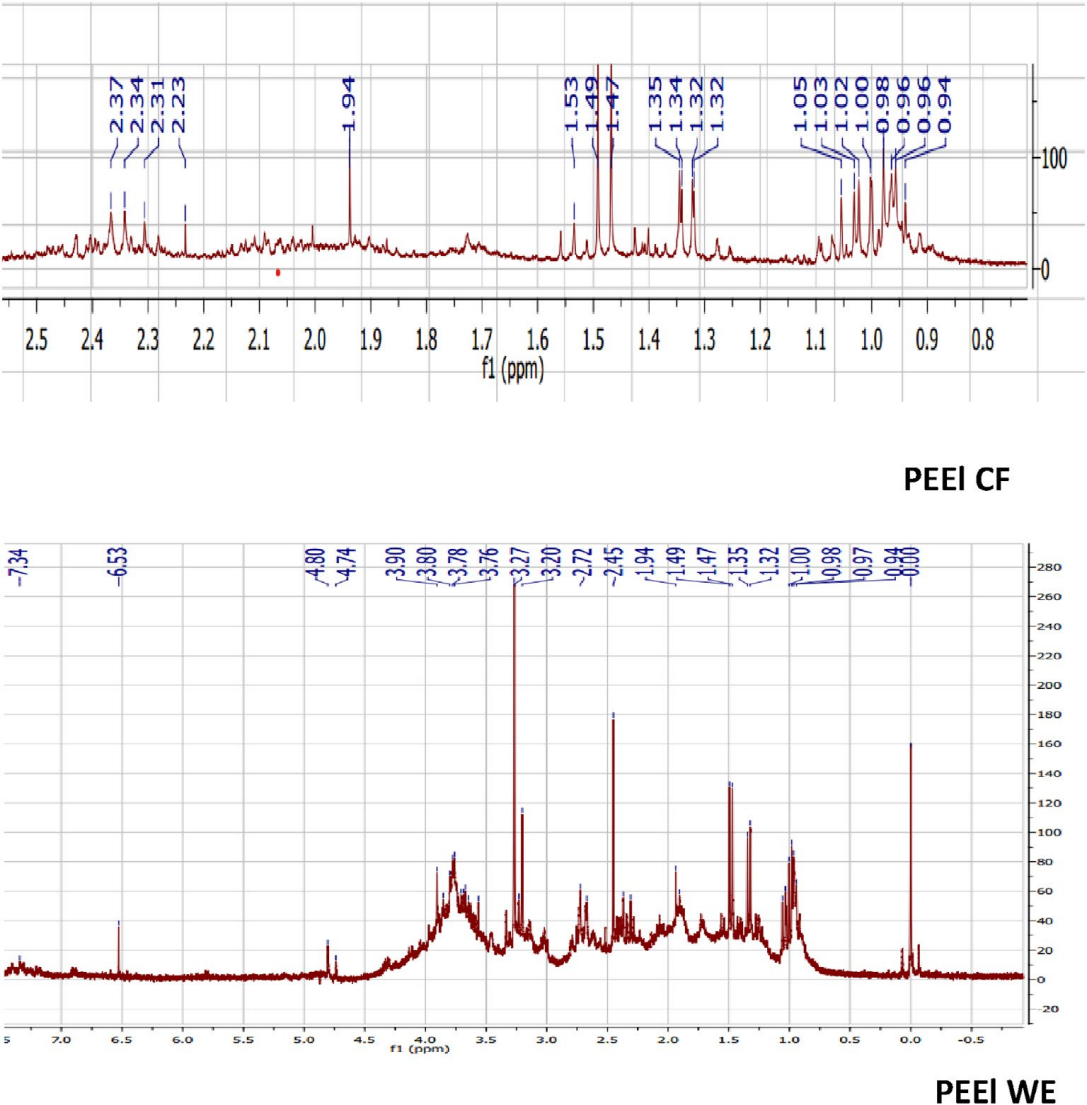
1H NMR analysis of *Pleurotus eryngii* var. *elaeoselini* mycelial culture filtrate (PEEl CF) and basidiocarps aqueous extracts (PEEl AE).

**FIGURE 6:**
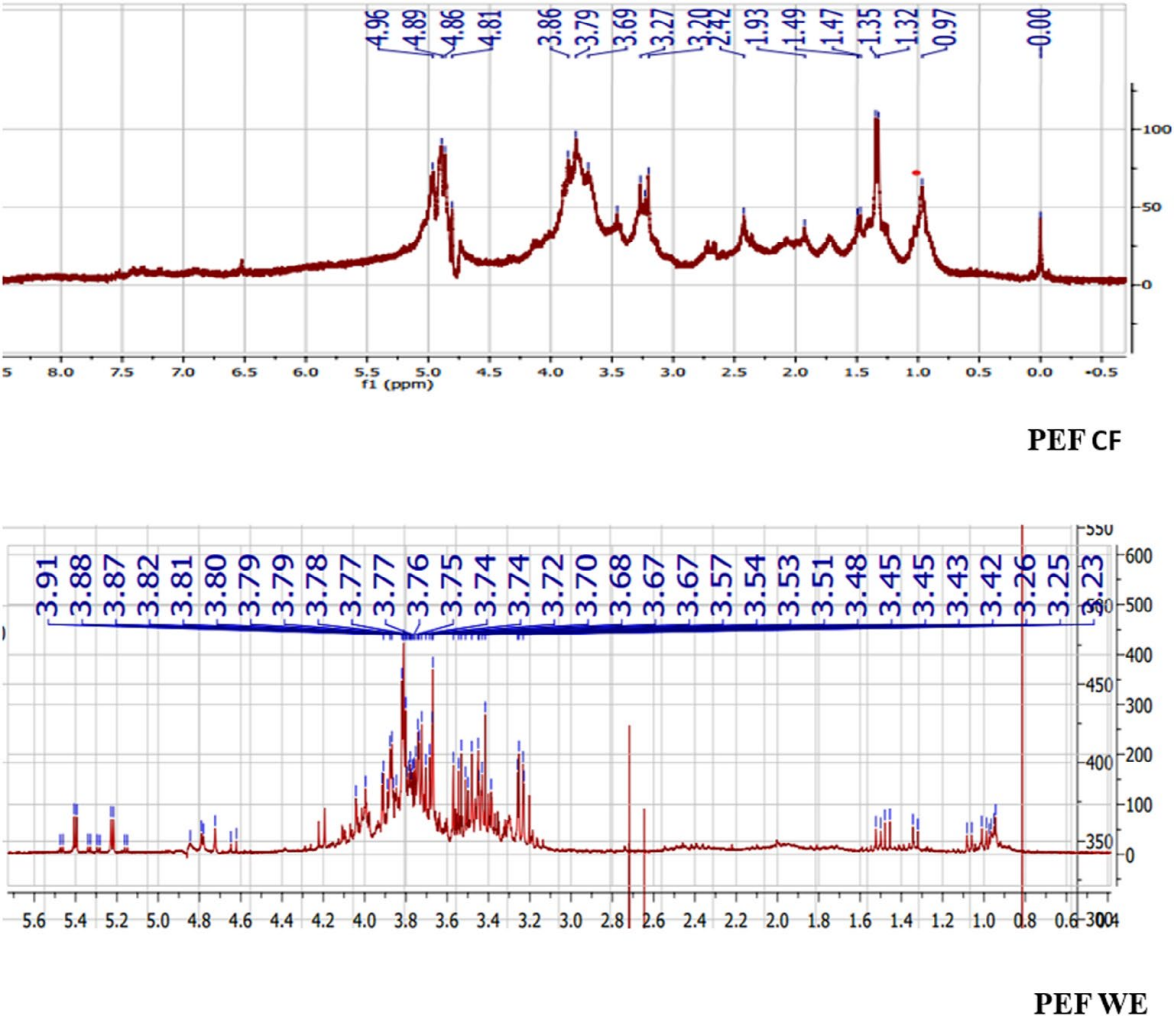
1H NMR analysis of *Pleurotus eryngii* var. *ferulae* mycelial culture filtrate (PEF CF) and basidiocarps aqueous extracts (PEF AE).

According to the 1H NMR spectra, most of the peak assignments in the mycelial culture filtrate and water extracts of the two varieties present pics of amino acids which range from 0.5 to 2 ppm. We note also the presence of organic acids from 2 to 3 ppm and carbohydrates from 3 to 5 ppm. These peaks are more pronounced in PEF culture filtrate (Figure 5) and water extracts (Figure 6), followed by PEEl water extracts. Peaks ranging from 6 to 8 ppm are also shown in PEEl water extract, which revealed the presence of alkaloids, flavonoids, and other secondary metabolites. In conclusion, all these samples are mixtures of proteins and carbohydrates and possibly small molecules like polyphenols.

## 4 Discussion

The aim of this study was to evaluate the ability of mycelial culture filtrates and aqueous extracts of carpophores isolated from the two oyster mushrooms to inhibit aflatoxin production by *A. flavus* . Tunisia has a hot climate and high relative humidity, which favors the growth of mycotoxin‐producing fungi and the synthesis of mycotoxins. PEEl metabolites inhibited the growth of *A. flavus* in the first days, while PEF did not seem to affect it. The growth of *A. flavus* was significantly impaired by the aqueous extracts of *Pleurotus eryngii* var. *elaeoselini* by 48.06% for the concentrations of 0.5 w/v, then by those of *Pleurotus eryngii* var. *ferulae*, whereas the growth of *Aspergillus flavus* was impaired by 27.35% at 0.5% w/v. Our results agree even if with different values of inhibition with those of Hussain and Hussein (2020), who found that methanolic extracts of *Pleurotus* varieties inhibited the growth of *Aspergillus* by 47.05% at a concentration of 250 ppm and that the growth reduction was “dose‐dependent” Moreover, a similar inhibition of growth of toxigenic strains by mushroom extracts was described by Zjalic et al. (2006) and Loncar et al. (2023). In both cases, growth inhibition was overcome over time, but aflatoxin inhibition persisted as for the PEF metabolites that did not significantly affect the growth of *A. flavus* , but significantly inhibited AFB1 production.

For the sake of clarity, current results indicate that the exo‐polysaccharidic fraction of these mushrooms is unable to act as antifungal; nonetheless, these compounds are formidable in blocking those mycotoxins which rely on a burst of oxidants to onset their biosynthesis (Scarpari et al. 2017). Moreover, lignin‐degrading mushrooms actively degrade aflatoxins through peroxidases and laccases action (Scarpari et al. 2014).

This study clearly shows a significant reduction of AFB1 by the two culture filtrates of the two varieties, with 94.5% for the two concentrations used in PEF and 86.95% in PEEl at the 1% w/v concentration. The inhibition is higher than that reported by Loncar et al. (2023). These authors showed that the two *P. eryngii* isolates studied reduced the aflatoxin content by 6.27% and 23.38%. Nevertheless, the same authors observed a high variability in aflatoxin inhibition potential between different isolates of the same species, suggesting that the inhibition potential might be related to the individual rather than being a trait of this species. However, Branà et al. (2017) reported that degradation of AFB1 by nine species of *Pleurotus* genera resulted in a reduction of 81%–99% after 10 days of growth. These authors also show that the genus *Pleurotus*, particularly *P. ostreatus* and *P. eryngii* , produces enzymes such as laccases and peroxidases that have been shown to be effective in neutralizing and degrading aflatoxins. In addition, treatment of AFB1 with manganese peroxidase from the white rot fungus *Phanerochaetes ordida* resulted in a detoxification of up to 86% of the initial level after 48 h. NMR analysis showed that both AE and CF are a mixture of amino acids, proteins, carbohydrates, and other molecules, and the presence of enzymes such as laccases or peroxidases cannot be excluded. Thus, the ability of *P. eryngii* to inhibit AFB1 production could result from a combination of effects, including differences in the biosynthetic kinetics of some enzymes involved in degradation, particularly laccases and Mn peroxidases (Motomura et al., 2003). According to Zjalic et al. (2006), the molecular analysis of two genes (aflR and norA) belonging to the aflatoxin cluster revealed an inhibition and a delay in their expression in the presence of *Trametes versicolor* culture filtrates. Moreover, it is known that aflatoxin biosynthesis is closely associated with oxidative stress and lipoperoxidation, and that this biosynthesis can be regulated by some antioxidants, which may play a crucial role (Reverberi et al. 2006; Mohsen et al. 2023). Indeed, our results show that anti‐aflatoxin activity may be related to antioxidant activity. The culture filtrate of *P. eryngii var. ferulae* has the highest AFB1‐inhibition ability as well as high antioxidant activity. Jaffali et al. (2024) showed that the PEF culture filtrate contains exopolysaccharides in the form of a mixture of α‐D‐mannan and mannogalactan. This mixture probably plays a role as peripheral structural components of the cell wall and extracellular secretions of *P. eryngii* . Both PEEl and PEF reduce aflatoxin synthesis, and their CFs and AEs have antioxidant capacity (DPPH assay), which could be associated with their richness in β‐D‐glucan (22.41%) (Jaffali et al., 2024). Loncar et al. (2023) show that fungal β‐D‐glucans could contribute to the inhibitory effect of aflatoxins by either acting as free radical scavengers or stimulating antioxidant enzymes in fungal cells. In addition, according to Gong et al. (2022), the polysaccharides obtained from *P. eryngii* are among the most important active ingredients and have a broad spectrum of biological activities.

Aqueous PEF extract of carpophore did not inhibit aflatoxin; instead, it enhanced its biosynthesis. This “opposite‐than‐expected” may be related to its high protein content of 17.69%. During aflatoxin biosynthesis, *A. flavus* efficiently assimilates amino acids such as methionine, phenylalanine, tyrosine, tryptophan, and acetate. In addition, the presence of nitrogen in the form of nitrite and nitrate enhances aflatoxin production by *A. flavus* through various mechanisms (Ahmad et al. 2022).

## 5 Conclusions

This study shows that water extracts and culture filtrates of the two Tunisian isolates of *P. eryngii* var. *elaeosini* and *P. eryngii* var. *ferulae* have the potential to limit the amount of aflatoxins produced in culture by *A. flavus* . NMR analysis showed that both extracts and filtrates are a mixture of organic compounds with a high content of proteins, amino acids, and carbohydrates, so the mechanism of action of their anti‐aflatoxin activity is not clear. It is probably a combination of multiple factors such as exopolysaccharides, which have antioxidant activity and can stimulate the antioxidant reaction in the cells of the toxigenic fungus, and aromatic‐degrading enzymes that can contribute to opening the heterocyclic lactone ring of AFB1, as demonstrated elsewhere (Zaccaria et al. 2023).

Further analysis of the extracts and filtrates is needed to better understand the mechanism of their action and the relationship between the different compounds and the inhibition/degradation of aflatoxins. This could enable the development of new natural anti‐aflatoxin compounds that are environmentally and economically sustainable.

## Author Contributions

**Chahrazed Jaffali:** formal analysis (equal), investigation (equal), methodology (equal), writing – original draft (equal). **Ayda Khadhri:** conceptualization (equal), formal analysis (equal), investigation (equal), supervision (equal), writing – original draft (equal), writing – review and editing (equal). **Marzia Beccaccioli:** methodology (equal), supervision (equal), writing – review and editing (equal). **Samira Aschi Smiti:** supervision (equal). **Massimo Reverberi:** methodology (equal). **Rosita Silvana Fratini:** writing – review and editing (equal). **Slaven Zjalic:** investigation (equal), methodology (equal), writing – review and editing (equal).

## Conflicts of Interest

The authors declare no conflicts of interest.

## Data Availability

All Data are available within the manuscript.
